# Green Tobacco Sickness among Brazilian farm workers and genetic polymorphisms

**DOI:** 10.1186/s13104-018-3135-x

**Published:** 2018-01-12

**Authors:** Marcelo Soares da Mota e Silva, Maria da Glória da Costa Carvalho, Josino Costa Moreira, Emiliano de Oliveira Barreto, Karol Fireman de Farias, Cristiane Araújo Nascimento, Francisca Maria Nunes da Silva, Tiago Gomes de Andrade, Ronir Raggio Luiz, Rodrigo Soares de Moura Neto, Fernanda Lattario Ribeiro

**Affiliations:** 1 Department of Pathology, Federal University of Rio de Janeiro, Rio de Janeiro, Brazil; 2 Center for the Studies on Workers’ Health and Human Ecology-Oswaldo Cruz Foundation, Rio de Janeiro, Brazil; 3 Biological and Health Sciences, Federal University of Alagoas, Maceió, Brazil; 4 Nursing School (Arapiraca Campus), UFAL, Arapiraca, Brazil; 5 Faculty of Medicine, UFAL, Maceio, Brazil; 6 Institute of Public Health Studies, UFRJ, Rio de Janeiro, Brazil; 7 Institute of Biology, UFRJ, Rio de Janeiro, Brazil

**Keywords:** Occupational health, Nicotine, Tobacco farm workers, Genetic polymorphisms

## Abstract

**Objective:**

Green Tobacco Sickness (GTS) is an occupational illness caused by dermal absorption of nicotine from tobacco leaves. It affects thousands of farm workers worldwide. Brazil is the second tobacco producer in the world; despite this, there are few studies on GTS among Brazilian harvesters. This study aimed to determine the prevalence of GTS among a population of tobacco workers from a producing area in northeastern Brazil and investigate whether the occurrence of the disease was influenced by factors such age, gender and smoking status. In addition, it was investigated if there was association between the onset of GTS and genetic polymorphisms in genes that encode some detoxification enzymes. A semi-structured questionnaire was used to collect demographic, behavioral and occupational data from the referred workers. Polymorphisms were tested through the Polymerase Chain Reaction technique.

**Results:**

The total prevalence of GTS found was 56.9%, with a significant difference between genders (71.7% for women and 35.3% for men, *p* < 0.0001). No association was identified between the investigated polymorphisms and GTS. This study confirms the occurrence of GTS among tobacco harvesters in Brazil with high prevalence. The investigation suggests the need to take preventive measures to protect tobacco workers against this disease.

## Introduction

Green Tobacco Sickness (GTS) is an acute poisoning caused by transdermal absorption of nicotine. It can affect tobacco farm workers as they come into contact with green tobacco leaves [1–3]. The most common symptoms are: dizziness, headache, muscle weakness, nausea and vomiting [1, 3, 4]. Visual changes have also been described in association with GTS [5]. Generally, the disease is relieved without medication. However, severe cases require hospitalization for treatment [6]. GTS is an important public health issue in tobacco-producing countries [7]. There are studies reporting the occurrence of the disease in several nations including Brazil [2–4, 6–8]. There are many risk factors linked to GTS such as contact with moist tobacco leaves (nicotine is water soluble), failure to use personal protective equipment during harvest, lack of experience in tobacco work and presence of skin cuts or rashes, [1, 2, 9]. It has been suggested in the literature that smoking can reduce the risk of GTS [1, 3]. The metabolism of nicotine is mediated by detoxification enzymes [10–12]. Polymorphisms in genes that encode these enzymes can decrease the efficiency or even prevent enzyme expression [13, 14]. This could impair the metabolism of nicotine.

This study aimed to investigate the occurrence of GTS in tobacco harvesters of northeastern Brazil. The prevalence of the disease was determined. The research investigated whether the occurrence of GTS was influenced by age, gender, smoking status, schooling and experience in tobacco fields. The study also analyzed polymorphisms in genes that encode the detoxification enzymes cytochrome P450 1A1 (CYP1A1), Glutathione *S*-transferase Mu 1 (GSTM1) and Glutathione *S*-transferase Theta 1 (GSTT1).

## Main text

### Methods

#### Study design and participants

This is a cross-sectional study. A non-random sample of 167 participants was recruited in four tobacco cultivation sites located in the rural area of Arapiraca, State of Alagoas, northeastern Brazil. The study included harvesters over the age of 18, who worked in tobacco fields, resided in the locality where the research was conducted and agreed to participate of the investigation. Data collection occurred between August and September 2011, at the time of tobacco harvest. A standardized semi-structured questionnaire was used to collect demographic, socio-economic, behavioral and occupational data from participants as well as to know the presence or absence of GTS symptoms. The questionnaire was conducted in a personal interview held at the selected tobacco cultivation sites.

#### DNA extraction and genotype analysis

For *GSTM1* and *GSTT1* genes, the polymorphism investigated was deletion, which represents the absence of gene sequence [13]. For *CYP1A1*, two polymorphisms were studied: m1 and m2. The former is a base substitution of thymine by cytosine at position 3801 [15]. It is referred as CYP1A1*2A, rs4646903 or 3801T > C [16]. The latter is a one base substitution A:T → G:C (adenine to guanine) at position 2455 in the exon 7 [15]. It is referred as CYP1A1*2C, rs1048943 or 2455A > G [16]. DNA from the subjects’ whole blood was extracted by using “FlexiGene^®^” kit (Qiagen, Hilden, Germany), Cat No./ID: 51206, adopting the manufacturer’s protocol. Polymorphisms in *CYP1A1*, *GSTM1* and *GSTT1* genes were detected through Polymerase Chain Reaction (PCR) technique, followed by polyacrylamide gel electrophoresis. DNA amplification was made with an initial DNA denaturing step at 95 °C for 5 min, followed by 30 cycles of 95 °C for 30 s, 64 °C for 1 min and 72 °C for 1 min. This was followed by a final extension of 72 °C for 5 min. The following primers were used:

- *CYP1A1 m1*-Fwd 5′-CAGTGAAGAGGTGTAGCCGCT-3′; *CYP1A1 m1*-Rev 5′-TAGGAGTCTTGTCTCATGCCT-3′
- *CYP1A1 m2*-Fwd 5′-GAAAGGCTGGGTCCACCCTCT-3′; *CYP1A1 m2*-Rev 5′-CCAGGAAGAGAAAGACCTCCCAGCGGGCCA-3′
- *GSTM1*- Fwd 5′-GAACTCCCTGAAAAGCTAAAG C-3′; *GSTM1*-Rev 5′-GTT GGGCTCAAATATACGGTGG-3′
- *GSTT1*- Fwd 5′-TTCCTTACTGGTCCTCACATCTC-3′; *GSTT1*-Rev 5′-TCA CCGGATCATGGCCAGCA-3′

The amplification of the investigated genes was performed at thermal cycler Mastercycler gradient^®^ from Eppendorf.

To investigate *CYP1A1* polymorphisms, in addition to PCR, restriction enzymes were used. *MspI* enzyme (New England Biolabs, USA, Cat # R0106S) was used to analyze m1 polymorphism and *NcoI* (New England Biolabs, USA, Cat # R0193S) for m2. *MspI* digestion produced 200 and 140 base pairs (bp) bands or 340, 140 and 200 bp bands, to homozygous and heterozygous polymorphic genotypes respectively, and an undigested band of 340 bp for *CYP1A1* wild type genotype. The *NcoI* digestion produced a 232 bp band for the wild type genotype, and two bands (232 and 263 bp) for m2 polymorphism. For *GSTM1* and *GSTT1*, the absence of the gene band (220 bp for *GSTM1* and 450 bp for *GSTT1*) represented its deletion. The methodology used for genotyping was previously described by Joseph et al. [17].

#### Statistical analysis

The Fisher exact test (two tailed) was used to compare the two groups (with and without GTS symptoms) according to *CYP1A1*, *GSTM1* and *GSTT1* genotypes. To determine the association between genotypes and age, gender, smoking status, experience in tobacco work and schooling, it was used the Fisher exact test (two tailed). The odds ratios and 95% confidence intervals were calculated and *p* < 0.05 was considered statistically significant. The software SPSS version 24.0 was used to make the statistical calculations.

### Results

The study sample included 167 participants, 68 men and 99 women; aged 19–78 years (41.3 ± 11.4 years). According to the completed questionnaires resulting from data collected in the personal interviews, tobacco harvest was completely manual at the study sites. The harvesters were in contact with leaves several hours a day. They did not wear gloves, boots or aprons. Among farm workers, 95 reported at least two concomitant GTS symptoms, reaching a total prevalence of 56.9% (CI 95%: 49.3–64.2). Separating by gender, 24 of 68 men had GTS, a prevalence of 35.3%. Among women, 71 of 99 had the disease, a prevalence of 71.7%. This difference between genders was highly statistically significant (*p* < 0.0001). The mean ± standard deviation age for workers with GTS was 40.7 ± 9.7 and 42 ± 13.2 years for workers without the disease. Even when divided into age groups, no significant difference was observed between those who presented the disease and those who did not present it (Table 1). Regarding the smoking status of the participants, 36 (21.6%) were smokers and 131 (78.4%) were not. Among the 95 workers who presented GTS, 17 (17.9%) were smokers and 78 (82.1%) were not. Of those who did not present the disease, 19 (26.4%) were smokers and 53 (73.6%) were not. However, this difference was not statistically significant (*p* = 0.25). Considering the variable “years worked in tobacco”, the prevalence of GTS did not show any significant difference among the workers of the distinct ranges adopted (Table 1). Likewise, schooling did not influence the occurrence of the disease in the study population.

**Table 1:** Characteristics of farm workers and Green Tobacco Sickness (GTS)

Variable	All workers N = 167	GTS yes N = 95	GTS no N = 72	*p* value	Unadjusted odds ratio (CI 95%)
	n (%)	n (%)	n (%)		
Gender				< 0.0001	
Male	68 (40.7)	24 (25.0)	44 (61.0)		0.21 (0.11–0.42)
Female	99 (59.3)	71 (75.0)	28 (39.0)		1
Smoking status				0.25	
Smoker	36 (21.6)	17 (17.9)	19 (26.4)		0.61 (0.29–1.27)
Non smoker	131 (78.4)	78 (82.1)	53 (73.6)		1
Age groups (years)				0.33	
19–38	70 (41.9)	41 (43.15)	29 (40.3)		1
39–58	88 (52.7)	51 (53.7)	37 (51.4)		0.97 (0.51–1.84)
59–78	9 (5.4)	3 (3.15)	6 (8.3)		0.35 (0.08–1.53)
Years worked in tobacco				0.082	
Up to 10	16 (9.6)	8 (8.4)	8 (11.1)		1.72 (0.50–5.9)
11–20	32 (19.2)	21 (22.1)	11 (15.3)		3.29 (1.16–9.34)
21–30	61 (36.5)	40 (42.1)	21 (29.2)		3.29 (1.32–8.18)
31–40	28 (16.8)	15 (15.8)	13 (18.0)		1.99 (0.69–5.69)
Above 40	30 (17.9)	11 (11.6)	19 (26.4)		1
Schooling (years)				0.125	
0–4	107 (64.1)	59 (62.1)	48 (66.7)		0.61 (0.05–6.98)
5–8	27 (16.2)	12 (12.6)	15 (20.8)		0.4 (0.03–4.95)
9–11	30 (17.9)	22 (23.2)	8 (11.1)		1.37 (0.11–17.3)
Above 11	3 (1.8)	2 (2.1)	1 (1.4)		1

Regarding the tasks carried out, most of the women participating in this study (73 of 99) performed the stalk removal of tobacco leaves. This task requires direct contact of the worker palms with leaves several hours a day. On the other hand, men did not participate in this activity.

The farm workers’ genotypic profile for *CYP1A1* (m1 and m2 polymorphisms), *GSTM1* and *GSTT1* genes was determined, as well as its association with GTS (Table 2). Regarding *CYP1A1,* the difference between frequencies of m1 and m2 polymorphisms among workers with or without GTS was of a maximum of 2%, but with no statistical significance (*p* = 0.87 and *p* = 1.0, respectively). Regarding *GSTM1* and *GSTT1*, the difference in the frequency of deletion was about 10%, but with no significance (*p* = 0.21 and *p* = 0.18, respectively).

**Table 2:** *CYP1A1*, *GSTM1* and *GSTT1* genotypes and Green Tobacco Sickness (GTS)

Genotypes	All workers N = 167 (%)	GTS yes N = 95 (%)	GTS no N = 72 (%)	*p* value
*CYP1A1* (m1 −)	104 (62.3)	60 (63.2)	44 (61.1)	0.872
*CYP1A1* (m1 +)	63 (37.7)	35 (36.8)	28 (38.9)	
*CYP1A1* (m2 −)	38 (22.8)	22 (23.2)	16 (22.2)	1.000
*CYP1A1* (m2 +)	129 (77.2)	73 (76.8)	56 (77.8)	
*GSTM1* present	89 (53.3)	55 (57.9)	34 (47.2)	0.210
*GSTM1* null	78 (46.7)	40 (42.1)	38 (52.8)	
*GSTT1* present	130 (78.0)	70 (73.7)	60 (83.3)	0.187
*GSTT1* null	37 (22.0)	25 (26.3)	12 (16.7)	

*m1* *−* no m1 polymorphism, *m1* *+* m1 polymorphism

*m2* *−* no m2 polymorphism; *m2* *+* m2 polymorphism

### Discussion

In this study, a considerable difference was found in the prevalence of GTS between genders (35.3% for men and 71.7% for women, *p* < 0.0001). It is possible that this difference is related to the distinct tasks performed by men and women. Considering the tasks, 73 of 99 women performed the stalk removal of tobacco leaves, which demands a continuous contact of the workers’ palms (an area of intense sweating) with the leaf surface, providing considerable absorption of nicotine, solubilized by sweat. Conversely, men did not remove stalks, but picked the leaves directly from tobacco feet, breaking the stalk of each leaf at its base. This task demands less contact of palms with leaves. In a study conducted in India, Parikh et al. found 42.66 and 55.7% of prevalence for men and women, respectively (*p* = 0.0659). However, the division of tasks between genders was not explored in the Indian study [6]. A higher prevalence of GTS among women could also be related to biological gender differences. Females have a relatively larger dermal area of absorption in relation to their body volume when compared to males [18].

A small difference between mean ages of workers with and without GTS (40.7 ± 9.7 and 42 ± 13.2 years, respectively) was found in this research. It suggests that age was not directly associated with the etiology of disease. Regarding smoking status, 17 (17.9%) of 95 workers with GTS and 19 (26.4%) of 72 workers without the disease were smokers. However, this difference was not statistically significant (*p* = 0.25). Therefore, it seems to indicate that smoking was not directly related to the onset of the disease, differing from other works of the literature [1, 3]. The variable “years worked in tobacco” does not appear to be associated with the occurrence of GTS in the study population. Observing the ranges of years worked (Table 1), there is no significant difference between farm workers who developed the disease and the others. This suggests that experience in tobacco fields was not relevant to avoid the disease. Regarding schooling, no significant difference was found between the groups, suggesting that there was no association between the level of schooling and the occurrence of GTS.

Considering the analyzed genotypes, as it can be seen in Table 2, no statistically significant difference was found between harvesters with GTS and harvesters without the disease. This suggests that there was no association between the investigated polymorphisms and the occurrence of GTS in the study population. Despite this, once genetic polymorphisms have been associated with several diseases, it might be interesting in future studies to analyze other polymorphisms besides the four investigated in this research. This would help to identify factors that can influence susceptibility to disease.

## Conclusion

This research identified the occurrence of GTS in northeastern Brazil with a high prevalence (56.9%). It should arouse the attention of public health authorities regarding to prevention of this important occupational illness. This agrees with the guidelines of the Framework Convention on Tobacco Control (FCTC) of which Brazil is a signatory. The articles 17 and 18 of the FCTC refer to the development of economic viable alternatives to tobacco growers, and the prevention of the negative impacts of tobacco growing and by-products on the environment and the health of tobacco growers [19].

## Limitations

The main limitation of this study is that the diagnosis of GTS was based solely on self-reported symptoms. In future investigations it would be useful to include some biochemical diagnostic method. It would be interesting, for example, to dose cotinine which is the main metabolite of nicotine in humans.

## Abbreviations

GTS: Green Tobacco Sickness
CYP1A1: Cytochrome P450 1A1
GSTM1: Glutathione *S*-transferase Mu 1
GSTT1: Glutathione *S*-transferase Theta 1
PCR: Polymerase Chain Reaction
Bp: base pairs
SPSS: Statistical Package for the Social Sciences
FCTC: Framework Convention on Tobacco Control
CI: confidence interval
